# Designing better diffracting crystals of biotin carboxyl carrier protein from *Pyrococcus horikoshii* by a mutation based on the crystal-packing propensity of amino acids

**DOI:** 10.1107/S2059798317010932

**Published:** 2017-08-15

**Authors:** Kazunori D. Yamada, Naoki Kunishima, Yoshinori Matsuura, Koshiro Nakai, Hisashi Naitow, Yoshinori Fukasawa, Kentaro Tomii

**Affiliations:** a Artificial Intelligence Research Center, National Institute of Advanced Industrial Science and Technology (AIST), 2-4-7 Aomi, Koto-ku, Tokyo 135-0064, Japan; b Graduate School of Information Sciences, Tohoku University, 6-3-09 Aramaki-Aza-Aoba, Aoba-ku, Sendai 980-8579, Japan; c RIKEN SPring-8 Center, 1-1-1 Kouto, Sayo-cho, Sayo-gun, Hyogo 679-5148, Japan; d Biotechnology Research Institute for Drug Discovery, National Institute of Advanced Industrial Science and Technology (AIST), 2-4-7 Aomi, Koto-ku, Tokyo 135-0064, Japan

**Keywords:** protein crystallography, surface engineering, crystal packing, crystal contact engineering, X-ray diffraction

## Abstract

An alternative rational approach to improve protein crystals by using single-site mutation of surface residues is proposed based on the results of a statistical analysis using a compiled data set of 918 independent crystal structures, thereby reflecting not only the entropic effect but also other effects upon protein crystallization. This analysis reveals a clear difference in the crystal-packing propensity of amino acids depending on the secondary-structural class. To verify this result, a systematic crystallization experiment was performed with the biotin carboxyl carrier protein from *Pyrococcus horikoshii* OT3 (*Ph*BCCP). Six single-site mutations were examined: Ala138 on the surface of a β-sheet was mutated to Ile, Tyr, Arg, Gln, Val and Lys. In agreement with prediction, it was observed that the two mutants (A138I and A138Y) harbouring the residues with the highest crystal-packing propensities for β-sheet at position 138 provided better crystallization scores relative to those of other constructs, including the wild type, and that the crystal-packing propensity for β-sheet provided the best correlation with the ratio of obtaining crystals. Two new crystal forms of these mutants were obtained that diffracted to high resolution, generating novel packing interfaces with the mutated residues (Ile/Tyr). The mutations introduced did not affect the overall structures, indicating that a β-sheet can accommodate a successful mutation if it is carefully selected so as to avoid intramolecular steric hindrance. A significant negative correlation between the ratio of obtaining amorphous precipitate and the crystal-packing propensity was also found.

## Abstract 2

In order to improve the efficiency of protein crystallization, an alternative approach using the mutation of surface residues was devised based on the results of a statistical analysis of the crystal-packing propensity of amino acids. A systematic crystallization experiment validated the results of the statistical analysis.

## 1. Introduction   

X-ray crystallographic analysis is currently an important method for the determination of protein structures. Along with advances in structural genomics and other efforts, the methodology of structure determination has been very well established. However, obtaining good protein crystals that are suitable for accurate structural determination persists as a severe bottleneck hindering X-ray crystallographic analysis, especially with respect to biologically important target proteins. To overcome the difficulties associated with crystallization, various approaches for improving the quality of protein crystals have been devised and implemented. Aside from high-throughput screening approaches, which have been widely adopted (Luft *et al.*, 2014 ▸), approaches involving the introduction of a crystallization nucleant (Sugahara *et al.*, 2008 ▸), pH optimization (Meged *et al.*, 2008 ▸), the adjustment of precipitant concentration (Bode & Huber, 1978 ▸), the addition of carrier proteins (Smyth *et al.*, 2003 ▸), homologous DNA shuffling (Keenan *et al.*, 2005 ▸), synthetically symmetrizing proteins (Banatao *et al.*, 2006 ▸), chemical modification (Kobayashi *et al.*, 1999 ▸; Kurinov *et al.*, 2000 ▸; Rypniewski *et al.*, 1993 ▸), proteolytic digestion of proteins (Wernimont & Edwards, 2009 ▸) and crystal contact engineering (Mizutani *et al.*, 2008 ▸; Wine *et al.*, 2009 ▸) have been proposed.

Among these various approaches, a notably successful method is surface-entropy reduction (SER; Derewenda, 2011 ▸), in which surface-exposed lysine and glutamate residues, which have high side-chain entropy (SCE), are replaced by residues with lower SCE, such as alanine, aspartate or serine, to promote protein crystallization. The effectiveness of this method has been proven through experimentation and subsequent data analyses (Longenecker *et al.*, 2001 ▸; Mateja *et al.*, 2002 ▸; Derewenda, 2004*a* ▸,*b* ▸; Price *et al.*, 2009 ▸; Goldschmidt *et al.*, 2007 ▸; Loll *et al.*, 2014 ▸). The fundamental concept of the SER method is based on the idea that a surface-exposed residue with a high SCE hinders protein crystallization by impairing the formation of proper crystal-packing states because of inherent flexibility of the side chain that elicits an entropic penalty when it is trapped by crystal contacts. Therefore, the most suitable residue for replacement in the SER method is deduced to be a surface-exposed lysine, which is known to be the most frequently observed residue on a protein surface.

However, despite the success of the SER method, the concept entails controversial issues. Firstly, although the SCE of arginine and glutamine is almost equal to or more likely greater than that of lysine (Doig & Sternberg, 1995 ▸; Pickett & Sternberg, 1993 ▸; Avbelj & Fele, 1998 ▸; Creamer, 2000 ▸), some reports have described that the replacement of a surface-exposed lysine with arginine or glutamine in proteins improves protein crystallization (Czepas *et al.*, 2004 ▸; Anstrom *et al.*, 2005 ▸; Mizutani *et al.*, 2008 ▸). These experimental results imply that impairment of crystal contact formation is not solely attributable to the SCE of the residues. Therefore, it might be hypothesized that it is not always optimal to replace a solvent-exposed lysine with an alanine to improve crystallizability. Indeed, the results of some experiments support this argument. For instance, Anstrom and coworkers compared the results of replacing a solvent-exposed lysine in malate synthase G by alanine and glutamine (Anstrom *et al.*, 2005 ▸). Contrary to the SER concept, they found that glutamine, with an almost identical SCE to that of lysine, was more effective than alanine as the substituting residue. Similarly, in the case of the N-terminal type II cohesin, the improvement of crystallizability by a lysine-to-tryptophan substitution was more significant when compared with a lysine-to-alanine substitution (Wine *et al.*, 2009 ▸). Moreover, some room exists for discussion of the relation between secondary structure and the SER method. According to a web prediction server which suggests residue(s) to be mutated based on the SER method (Goldschmidt *et al.*, 2007 ▸), surface-exposed residues in coil regions are positively indicated as target residues for replacement, and residues in helices and strands are excluded as mutation targets. However, Anstrom *et al.* (2005 ▸) showed that the effect of the SER method did not change depending on whether the residues were in a coil, helix or strand. Therefore, the role of secondary structure in residue selection remains a topic of debate. Furthermore, if the effectiveness of mutation of residues in a helix or strand region on crystallization improvement is validated, then the range of choices of mutation residues will be expanded greatly because, for example, 56% of all lysine residues in proteins are found in helix or strand regions (Baud & Karlin, 1999 ▸).

Therefore, this study was conducted to improve the crystallizability of proteins using single-site mutation of surface residues, and to address the issues presented above. To this end, we have compiled a larger data set of protein crystal structures based on data sets used in previous studies. We calculated the crystal-packing propensities of 20 amino acids depending on three secondary-structure classes: helix, sheet and coil. We discovered distinctive propensities of 20 amino acids depending on these three classes. As a proof of concept of our statistical analysis, this finding was evaluated using a systematic crystallization experiment of a model protein, the biotin carboxyl carrier protein from *Pyrococcus horikoshii* OT3 (*Ph*BCCP), in which an alanine residue located in the β-sheet structure was replaced by residues of six other types: Ile, Tyr, Arg, Gln, Val and Lys. Using the seven constructs including the wild type, a crystallization screening experiment was performed, revealing that the hit rates in the crystallization screening were correlated with the crystal-packing propensities of the amino acids. Then, according to our hypothesis, we were able to obtain two new crystal forms that diffracted to high resolution from two of the mutants. The packing of these crystals was analyzed from the perspectives of various interaction properties. In light of the experimentally obtained results, we discuss the possibility of expanding the range of proteins that are applicable to X-ray crystallography using our approach.

## 2. Materials and methods   

### 2.1. Data set   

To conduct statistical analyses of crystal structures, we compiled a new data set by combining two data sets consisting of 821 (Cieślik & Derewenda, 2009 ▸) and 817 (Carugo & Djinović-Carugo, 2012 ▸) PDB entries that were used in previous studies. The resolutions of the protein structures in both data sets are 2.5 Å or better. The asymmetric unit of each entry is one molecule. We removed duplicate entries from these data sets and thereby obtained 1519 unique protein entries. We selected only the 1403 monomeric protein structures by checking the BIOLOGICAL UNIT record in the PDB files and the literature so that the protein–protein interfaces of each entry are ensured to be crystal contacts. We removed the redundancy within the data set by clustering proteins with a sequence identity of 40% using the *CD-HIT* package (Fu *et al.*, 2012 ▸), finally leading to 918 protein structures.

### 2.2. Definition of crystal contacts and secondary structures   

In this study, two protein molecules were regarded as neighbours if the minimum distance between an atom belonging to one molecule and an atom belonging to the other molecule was less than 5 Å. Based on this distance threshold, a complex of protein molecules comprising a molecule in the crystallographic asymmetric unit and its neighbours was generated for each PDB entry using the *MOE* software package (Chemical Computing Group). For each residue of the molecule in the asymmetric unit, the accessible surface area (ASA) and the ASA buried by crystal contacts were calculated using the *pro_ASAcalc* function from the *MOE* software package. A surface region of a protein molecule with a nonzero value of buried ASA was defined as a contact interface, whereas that with only a nonzero positive value of ASA was defined as a protein surface. The areas of contact interfaces (interface area) and those of protein surfaces (surface area) were summed separately in all PDB entries selected. The interface area and the surface area were used to calculate the crystal-packing propensity as described in the next section.

We assigned secondary-structure information to each residue of all proteins in the data set using the *DSSP* program (Kabsch & Sander, 1983 ▸). *DSSP* categorizes secondary structures into eight states. We divided these eight states into three secondary-structure classes: H, G and I (regarded as helix), B and E (regarded as sheet), and T, S and C (regarded as coil in this study).

### 2.3. Crystal-packing propensity   

We calculated the crystal-packing propensity of 20 amino acids and the three secondary-structure classes using the data set compiled as described above. As a crystal-packing propensity, we calculated and used the crystal-packing formation likelihood for each amino acid and each secondary-structure class. In other words, the crystal-packing propensity represents the likelihood of the involvement of an amino acid or a secondary-structure class of interest in the crystal contact. The likelihood *L_i_* of amino acid *i* (*i* = 1, 2, …, 20) (Supplementary Fig. S3) or secondary structure *i* (*i* = 1, 2, 3) is calculated as
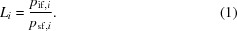
In (1), *p*_if,*i*_ and *p*_sf,*i*_ represent the ratio of the interface area of amino acid or secondary structure *i* to the total interface area and the surface area of amino acid or secondary structure *i* to the total surface area, respectively. We also separately calculated the likelihood of amino acid *i* (*i* = 1, 2, …, 20) for each secondary-structure class *s* (*s* = helix, sheet, coil):
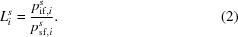
Regarding this indicator, a value of *L_i_* of greater than 1 signifies that amino acid or secondary structure *i* tends to be involved in crystal contact formation in a positive manner. A value smaller than 1 means the opposite. A value of 1 signifies that the residue has a neutral propensity for crystal contact formation.

### 2.4. Statistical tests   

The goodness-of-fit test for likelihood of crystal contact formation was analyzed using a one-sample Kolmogorov–Smirnov test, which is used to decide whether instances in a sample come from a specific distribution (Chakravarti *et al.*, 1967 ▸). We conducted the test against the null hypothesis where the distribution of the propensity was identical to a uniform distribution using the *ks.test* function in the *R* package.

The significance of the association between two variables was analyzed using Fisher’s exact probability test (Fisher, 1922 ▸) to decide whether two variables in a contingency table are independent of each other. Fisher’s exact probability test was conducted using the *fisher.test* function in the *R* package.

### 2.5. Mutagenesis and production of *Ph*BCCP   

Site-directed mutagenesis was performed using the QuikChange mutagenesis kit (Agilent Technologies Inc.). Protein expression and purification of the *Ph*BCCPΔN79 proteins (wild type, A138I, A138Y, A138R, A138Q, A138V and A138K) were performed as described in a previous report (Bagautdinov *et al.*, 2007 ▸) except that the last gel-filtration column used was Superdex 75 instead of Superdex 200. Purified samples showed single bands on SDS–PAGE (Supplementary Fig. S4). The protein concentration was determined using the Pierce 660 nm Protein Assay Reagent (Thermo Scientific) and was proportionally corrected based on the concentration of the A138Y mutant, which was determined spectrophoto­metrically using an absorption coefficient of 1470 *M* ^−1^ cm^−1^ at 280 nm. After concentration by ultrafiltration (Vivaspin, 3 kDa cutoff; GE Healthcare), the protein solution (12.3–13.5 mg ml^−1^ *Ph*BCCPΔN79, 0.2 *M* sodium chloride, 20 m*M* Tris–HCl pH 8.0) was stored at 277 K.

### 2.6. Crystallization and data collection   

Crystallization screening of the *Ph*BCCPΔN79 proteins was performed using the sitting-drop vapour-diffusion method with eight commercially available crystallization kits comprising 528 conditions in total: Crystal Screen and Crystal Screen 2 (96 conditions; Hampton Research), Index (96 conditions; Hampton Research), SaltRX (96 conditions; Hampton Research), PEG/Ion (96 conditions; Hampton Research), Wizard I and II (96 conditions; Rigaku) and Wizard III (48 conditions; Rigaku). The 0.4 µl crystallization drop was prepared by mixing equal volumes of protein solution and reservoir solution on a 96-well crystallization plate (VIOLAMO VCP-1; AS ONE) using a Mosquito nano­dispenser (TTP Labtech). Crystallization plates were stored in an imaging system (Crystal Farm CF-400; Discovery Partners International) at 293 K. Microscopic images of drops were taken one week, four weeks and six weeks after the start of the experiment. The images were classified manually into four categories: clear drop, amorphous precipitate, a single crystal with a minimum dimension of greater than 30 µm, referred to as ‘analyzable crystal’, and other crystals such as microcrystals, thin plate/needle crystals and clustered crystals, referred to as ‘non-analyzable crystal’. Note that this is a practical classification to analyze hundreds of crystallization drops efficiently, although a diffraction-based definition may be more rigorous. Crystallization for X-ray data collection was performed manually using the hanging-drop vapour-diffusion method with the same conditions as were used for crystallization screening. The 2.0 µl crystallization drop was prepared on a 24-well crystallization plate (VDXm; Hampton Research) by mixing equal volumes of protein solution and reservoir solution: 3.5 *M* sodium formate, 0.1 *M* Tris buffer pH 8.5 for the wild type, 20% PEG 3350, 0.2 *M* magnesium formate pH 7.0 for the A138I mutant and 2.5 *M* sodium chloride, 0.2 *M* lithium sulfate, 0.1 *M* acetate–NaOH pH 4.5 for the A138Y mutant. The crystallization plate was stored at 293 K. Orthorhombic rod-shaped crystals of the wild-type protein grew in 5 d to approximate dimensions of 100 × 500 × 100 µm. Orthorhombic plate-shaped crystals of the A138I mutant grew in two weeks to approximate dimensions of 300 × 500 × 100 µm. Thick trigonal crystals of the A138Y mutant grew in 5 d to approximate dimensions of 200 × 200 × 100 µm. X-ray diffraction data were collected using a CCD detector (MAR Mosaic 225) on the BL26B2 beamline at SPring-8, Japan. Data collection was performed at 100 K using flash-cooled crystals. The cryoprotectant solution used was 20%(*v*/*v*) glycerol in the reservoir solution. The diffraction data were processed and scaled using *HKL*-2000 (Otwinowski & Minor, 1997 ▸) and were converted to structure factors using the *CCP*4 program suite (Winn *et al.*, 2011 ▸). Details related to data collection are presented in Table 2 and Supplementary Table S2.

### 2.7. Determination and evaluation of crystal structure   

The *Ph*BCCPΔN79 crystal structure was determined using *PHENIX* (Adams *et al.*, 2002 ▸). Wild-type *Ph*BCCPΔN76 (PDB entry 2evb; Bagautdinov *et al.*, 2008 ▸) was used as the search model for molecular replacement. The structure was visualized using *Coot* (Emsley & Cowtan, 2004 ▸). For comparison of the effective resolution between data sets, the resolution limit of each data set was adjusted at the last stage of structure refinement so that the *R*_free_ value for the outmost shell was less than 30%. Statistics of the refinement are presented in Table 2 and Supplementary Table S2. Superposition of coordinates, calculation of accessible surface areas and analysis of interatomic distances were performed using programs from the *CCP*4 suite: *LSQKAB* (Kabsch, 1976 ▸), *SURFACE* (Lee & Richards, 1971 ▸) and *ACT* (Kabsch & Sander, 1983 ▸), respectively.

## 3. Results   

### 3.1. Crystal-packing propensity of secondary structures   

**Figure 1:**
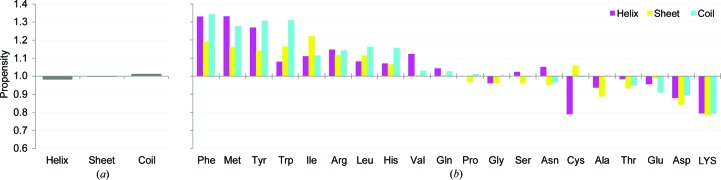
Crystal-packing propensity. (*a*) Crystal-packing propensity of secondary structures. The crystal-packing propensity, *i.e.* the crystal-packing formation likelihood *L* ^*s*^_*i*_, of three secondary-structure classes is shown: *i* = 1 (helix), 2 (sheet), 3 (coil). See §§[Sec sec2.2]2.2 and [Sec sec2.3]2.3 for definitions of *L* ^*s*^_*i*_ and the three secondary-structure classes. (*b*) Crystal-packing propensity of amino acids depending on the secondary-structure class. The crystal-packing propensity, *i.e.* the crystal-packing formation likelihood *L* ^*s*^_*i*_, of 20 amino acids for three secondary-structure classes is shown: helix (magenta), sheet (yellow) and coil (cyan).

Firstly, using the newly compiled data set, we calculated the crystal-packing propensities for the three secondary-structure classes helix, sheet and coil (see §[Sec sec2.2]2.2 for definitions) to ascertain whether a difference exists in the crystal contact formation propensity. Fig. 1 ▸(*a*) presents a comparison of the propensity for the three secondary-structure classes. As a result, the crystal-packing propensities of the three structure classes were found to be almost identical. Indeed, it was concluded that the difference was not of statistical significance according to a one-sample Kolmogorov–Smirnov test against a null hypothesis that the distribution of the crystal-packing propensity of the secondary structures was identical to a uniform distribution (*p* > 0.05). This suggests that the occurrence of crystal contacts is invariable in terms of secondary structure. In turn, this implies that it is not necessary to limit amino-acid substitution to coil regions to improve protein crystals, as proposed in a previous study (Cooper *et al.*, 2007 ▸; Derewenda, 2004*a* ▸,*b* ▸). In addition, this observation is consistent with the results reported by Anstrom *et al.* (2005 ▸). However, a possible reason for selecting loop regions in the SER strategy is that mutations in these parts of the protein are less likely to perturb the overall structure.

### 3.2. Crystal-packing propensity of amino acids in different secondary structures   

The results showed that crystal contacts can occur irrespective of the secondary-structure class. Therefore, it is expected to be highly beneficial to exploit all surface residues of a protein to form crystal contacts and to improve the protein crystal. Using the newly compiled data set, we calculated the crystal-packing propensities of 20 amino acids depending on the three secondary-structure classes. Fig. 1 ▸(*b*) presents the results of a comparison of the propensity of each amino acid in different secondary structures. These results showed that the propensity of lysine was almost identical irrespective of the secondary-structure class. The results also confirmed that lysine is the residue with the worst crystal-packing propensity, as was widely believed. We note that there were differences and characteristics of the propensities of some amino acids depending on the secondary structure. The propensities of some amino acids as deduced based on a structural class and combining all structural classes were found to differ distinctly. We confirmed this point by sampling shuffled data sets (Supplementary Fig. S1). For instance, valine on a helix tended to be more involved in crystal contacts than on sheets and coils. However, isoleucine on a sheet was more preferred in crystal contact formation than isoleucine on a helix or coil. As a possible explanation for this observation, especially for the case of valine on a helix, we speculate on the well known relation between α-helix-formation propensity and side-chain entropy of amino acids (Creamer & Rose, 1994 ▸; Chellgren & Creamer, 2006 ▸). Valine has the highest rank order of entropy loss for helix formation. However, no entropy loss occurred when valine on a helix formed crystal contacts, although side-chain entropy loss might occur when valine on a sheet or coil participates in crystal contacts because freedom of the rotamers of valine on a sheet or coil is permitted. As described above, the crystal-packing propensity of amino-acid residues can vary depending on the secondary-structure class. Consequently, using the propensity calculated here, one can suggest a desirable replacement of a surface residue depending on the secondary structure.

### 3.3. Experiment design   

As a systematic crystallization experiment to validate the results of our statistical analysis, we selected the biotin carboxyl carrier protein from *P. horikoshii* OT3 (*Ph*BCCP) as a model system. We previously determined the crystal structure of the C-terminal 73-residue fragment of *Ph*BCCP (*Ph*BCCPΔN76) that represents the biotinyl(/lipoyl attachment) domain of the hypothetical methylmalonyl-CoA decarboxylase γ chain (Bagautdinov *et al.*, 2008 ▸). This domain adopts a flattened β-barrel comprising two homologous ‘hammerhead’ structures that confer an intramolecular pseudo-twofold symmetry reflecting the gene duplication (Athappilly & Hendrickson, 1995 ▸). *Ph*BCCPΔN76 is a small monomeric protein with no disordered region and with moderate crystallizability, allowing a strong influence of single-site mutation on crystallizability and uncomplicated interpretation of the experimental results. Full-length *Ph*BCCP has large disordered regions in its N-terminal half, which are not appropriate as a model system. In this work, we used the C-terminal 70-residue fragment of *Ph*BCCP (*Ph*BCCPΔN79) as the template to prevent the N-terminal heterogeneity that was observed in *Ph*BCCPΔN76. In the *Ph*BCCP system (*Ph*BCCPΔN79) residue 138 was selected as the target residue for the mutation experiment because it is a solvent-exposed and nonconserved residue on a surface β-sheet. Moreover, in modelling it could be replaced by any other residue except for proline without intramolecular steric hindrance. Considering the balance of crystal-packing propensity for β-sheet (Table 1 ▸) and comparison with the SER method, we designed a systematic site-directed mutagenesis substituting Ala138 with residues of six other types: Ile (the highest crystal-packing propensity), Tyr, Arg, Gln, Val and Lys (the lowest propensity).

### 3.4. Crystallization screening   

Crystallization screening with a drop volume of 0.4 µl was performed for six weeks at 293 K for the wild type and six mutants of *Ph*BCCPΔN79 using eight commercially available crystallization kits comprising 528 conditions in total. As a result, a clear difference was observed in the distribution of crystallization scores depending on the type of residue at position 138 (Table 1 ▸). The isoleucine mutant (A138I) and the tyrosine mutant (A138Y) conferring residue 138 with the highest crystal-packing propensities for β-sheet provided better crystallization scores when compared with other constructs, including the wild type. The other mutants A138R, A138Q and A138V with moderate crystal-packing propensities yielded scores similar to those for the wild type. The crystallization scores for the A138K mutant with the lowest crystal-packing propensity were the worst of all of the constructs, as anticipated.

### 3.5. Correlations between crystallization results and crystal-packing propensity   

**Figure 2:**
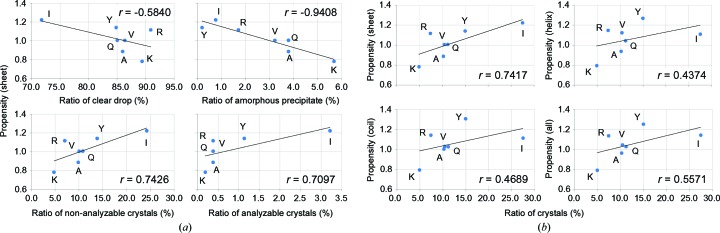
Statistical analysis of crystallization results. (*a*) Correlation between crystallization results and crystal-packing propensity for β-sheet. For each *Ph*BCCPΔN79 construct, ratio values of four categories, *i.e.* the ratios of clear drop (top left), amorphous precipitate (top right), non-analyzable crystal (bottom left) and analyzable crystal (bottom right), from the crystallization screening experiment (horizontal axis) are plotted *versus* the crystal-packing propensity of residue 138 (vertical axis) with overlaid linear regressions. Labelling for each dot denotes the single-letter code of residue 138. The Pearson correlation coefficient (*r*) is given in each graph. (*b*) Correlation between crystal-packing propensity and ratios of crystals. The ratio of obtaining crystals from the crystallization screening experiment was calculated as (number of non-analyzable crystals + analyzable crystals) × 100/528. The ratios of crystals (horizontal axis) are plotted *versus* the crystal-packing propensity of residue 138 (vertical axis) in the same manner as that shown in (*a*), except that the crystal-packing propensities of four types calculated from different secondary structures are compared: sheet (top left), helix (top right), coil (bottom left) and all (bottom right).

To elucidate the influence of mutation upon crystallization, the correlation between crystallization results and crystal-packing propensity was analyzed (Fig. 2 ▸ *a*). The ratios of obtaining non-analyzable and analyzable crystals (see §[Sec sec2.6]2.6 for definitions) showed strong positive correlation with the crystal-packing propensity of amino acids for β-sheet; the Pearson correlation coefficients (*r*) were 0.74 (*p* = 0.056) and 0.71 (*p* = 0.074), respectively. The ratio of obtaining crystals that was calculated from the summation of non-analyzable and analyzable crystals showed the best correlation *r* = 0.74 (*p* = 0.056) with the propensity for β-sheet, whereas the *r* values were 0.44 (*p* = 0.33) for helix, 0.47 (*p* = 0.29) for coil and 0.56 (*p* = 0.19) for all three structure classes (Fig. 2 ▸ *b*). It is noteworthy that the packing propensity from β-sheet only can account for the order of observed crystallizability from the experiment, *i.e.* Ile > Tyr > Val.

Two other ratio values from the crystallization results correlated negatively with the crystal-packing propensity for β-sheet (Fig. 2 ▸ *a*). It is particularly interesting that a very strong negative correlation was found between the ratio of obtaining amorphous precipitate and the crystal-packing propensity, with an *r* value of −0.94 (*p* = 0.0016), which was the strongest correlation among the present crystallization results. From Fisher’s exact probability test, it was statistically proved that a construct harbouring a residue 138 with a crystal-packing propensity of higher than 1.04 provides a ratio of obtaining amorphous precipitate of lower than 2.5%, using a significance level of 5% (*p* = 0.0286; Supplementary Table S1). In addition, a moderate correlation *r* = −0.58 (*p* = 0.17) was found between the ratio of obtaining a clear drop and the packing propensity.

### 3.6. Crystal structures of the wild type and mutants   

**Figure 3:**
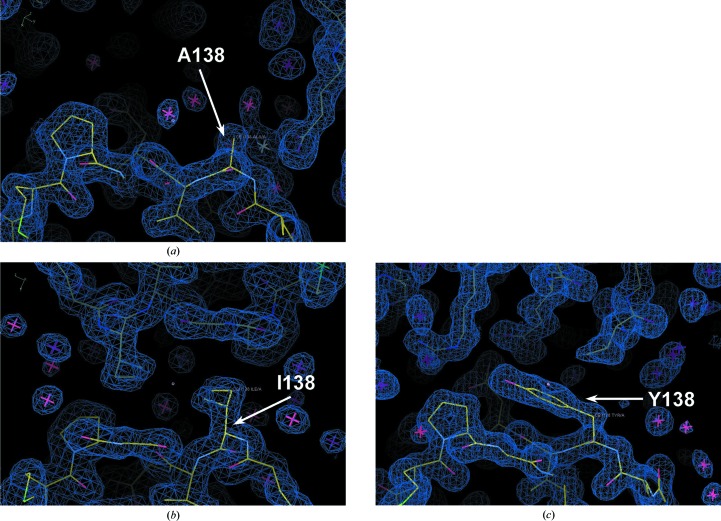
Structure of *Ph*BCCPΔN79 crystals around residue 138. Protein molecules are depicted as stick models with atom-type colouring in (*a*) the wild type, (*b*) the A138I mutant and (*c*) the A138Y mutant. The 2*F*_o_ − *F*_c_ electron-density maps from refined models contoured at 1.0σ are overlaid. Red and white asterisks denote water molecules and a sodium ion (in the wild type), respectively. C atoms in the asymmetric unit molecule and those in the symmetry-related molecule are distinguished by yellow and grey colouring, respectively. This figure was prepared using *Coot*.

To explore the structure–mutation relation, we determined the structures of the *Ph*BCCPΔN79 crystals obtained from three constructs: wild type, A138I and A138Y (Table 2 ▸ and Supplementary Table S2). These three crystals were selected because they diffracted X-rays well and were crystallized from unique crystallization conditions. The condition that was found to be suitable for the crystallization of one construct did not provide analyzable crystals for the other constructs. In all three crystal forms the crystallographic asymmetric unit contained a *Ph*BCCPΔN79 monomer, although the modes of crystal packing mutually differed. The wild-type protein produced an orthorhombic crystal form belonging to space group *P*2_1_2_1_2_1_. The structure was determined at a resolution of 1.8 Å, which was intermediate in quality when compared with those of other structures. Probably because of the deletion of three N-terminal residues, the crystal-packing mode obtained differs from that of the reported *Ph*BCCPΔN76 crystal (PDB entry 2evb), although they share the same space group and their unit-cell parameters are similar. Residue 138 is located at the perimeter of a crystal-packing interface. The side chain of Ala138 is 68% exposed to the solvent (Fig. 3 ▸ *a* and Supplementary Fig. S2*a*). The A138I mutant and the A138Y mutant produced another orthorhombic crystal form belonging to space group *C*222_1_ and a trigonal crystal form belonging to space group *P*3_1_21, respectively. The crystal structures of the A138I and A138Y mutants were determined at resolutions of 1.9 and 1.5 Å, respectively. In these mutant crystals the crystal-packing interface involving residue 138 differs completely from those of the other two crystal forms. Also, the side chain of residue 138 is about 80% buried at each interface (Figs. 3 ▸ *b* and 3 ▸ *c* and Supplementary Figs. S2*b* and S2*c*). Neither the *C* orthorhombic nor the trigonal crystals could be obtained with the wild-type protein. Therefore, it is conceivable that the substitution of Ala by Ile/Tyr with a better crystal-packing propensity created novel packing interfaces.

**Figure 4:**
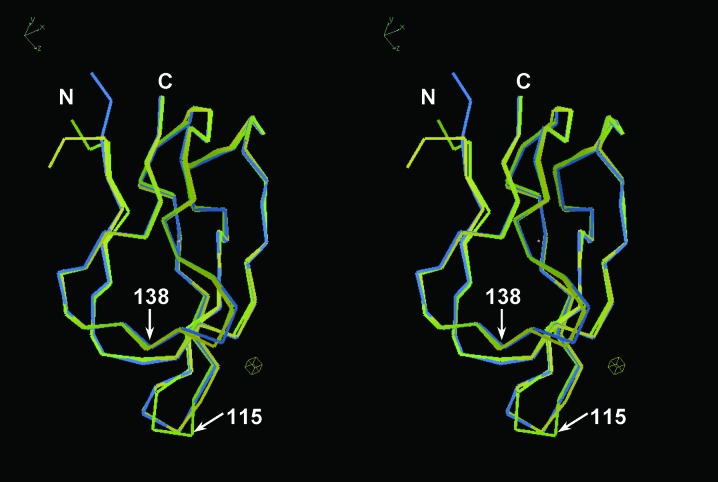
Stereoview of superimposed overall structures of *Ph*BCCPΔN79 crystals. Protein molecules in the asymmetric unit are superimposed at corresponding C^α^ atoms and are depicted as C^α^ models in different colours: wild type in yellow, A138I in green and A138Y in blue. The N-­terminus and C-terminus and residues 115 and 138 are labelled. This figure was prepared using *Coot*.

The similarity of the three crystal structures was confirmed using structural superposition. The two N-terminal residues were excluded from the superposition because they adopted completely different conformations (Fig. 4 ▸), reflecting the difference in crystal packing. After superposition, the root-mean-square deviation (r.m.s.d.) of the interatomic distances of corresponding C^α^ atoms (residues 81–149; 69 pairs) was in the range 0.35–0.57 Å (Supplementary Table S3). Moderate deviations of up to 2.53 Å were observed at residues 114–116, which constitute a flexible β-turn harbouring the Lys115 residue to be biotinylated (Bagautdinov *et al.*, 2008 ▸). Therefore, the overall structures of *Ph*BCCPΔN79 trapped in different crystal-packing modes were essentially the same apart from the N-terminus and the flexible β-turn (Fig. 4 ▸).

### 3.7. Relation between crystal-packing interactions and X-ray diffraction resolution   

We analyzed the crystal-packing interactions of the three crystal structures to investigate the factors contributing to the X-ray diffraction resolution of the crystals. To characterize the packing mode of each crystal form, the crystal-packing interactions were classified as one of four types and were counted: nonpolar interactions, hydrogen bonds, indirect polar interactions mediated by a water/ion molecule and electrostatic interactions (Table 3 ▸). The wild-type crystal packing is rich in indirect polar interactions mediated by water molecules and a sodium ion, as observed in many other protein crystals (Carugo & Djinović-Carugo, 2014 ▸). On the other hand, the crystal packings of the A138I and A138Y mutants are rich in attractive electrostatic interactions and nonpolar interactions, respectively. It is particularly interesting that of these types of interactions, only nonpolar interactions, *i.e.* van der Waals interactions, can account for the diffraction limit of crystals. The numbers of nonpolar interactions normalized by the total buried ASA (Supplementary Table S6) for the three crystal forms were calculated as 0.108 Å^−2^ for the wild type, 0.099 Å^−2^ for the A138I mutant and 0.135 Å^−2^ for the A138Y mutant. The order of the normalized number of nonpolar interactions corresponds to that of the resolution. In other words, the density of nonpolar interactions at the crystal-packing interface might be a factor that affects the diffraction quality of protein crystals in the *Ph*BCCP system.

## 4. Discussion   

To improve the crystallization success rate and the crystal quality of a protein, methods in which the surface residues of the protein are replaced with other residues have been commonly used in X-ray diffraction studies. Among surface-engineering approaches, the SER method has been widely used, with the aim of reducing the ‘entropic shield’ on the protein surface. In contrast, we have proposed an alternative rational approach to improve protein crystals by using single-site mutation of surface residues based on the results of a statistical analysis using a compiled data set of 918 independent crystal structures, thereby reflecting not only the entropic effect but also other effects upon protein crystallization. Our approach includes the use of a ‘sticky’ or ‘enthalpically favoured’ single-site mutation of surface residue(s) based on the results of statistical analysis, *i.e.* the distinctive crystal-packing propensity of amino acids depending on three secondary-structure classes.

To examine and assess our approach, we conducted a systematic crystallization experiment using the *Ph*BCCP system. We confirmed that the experimentally obtained results of the crystallization screening show good agreement with predictions based on the crystal-packing propensity. The propensity for β-sheet provided the best correlation with the ratio of obtaining crystals, indicating that the crystal packing involving secondary-structural elements was important (Fig. 2 ▸ *b*). However, the success rate of obtaining crystals for the A138R mutant was low when compared with that expected from the crystal-packing propensity; this might be a characteristic of the *Ph*BCCP system and should be examined further in other systems. A very strong negative correlation was found between the ratio of obtaining amorphous precipitate and the crystal-packing propensity (Fig. 2 ▸ *a*). In contrast to the argument that hydrophobic interactions engender disordered precipitates and not crystals (Dasgupta *et al.*, 1997 ▸), our results suggest that the introduction of a residue with a high crystal-packing propensity in the *Ph*BCCP system elicited specific intermolecular interactions and produced crystals instead of amorphous precipitates.

The crystal structures of *Ph*BCCPΔN79 from three constructs, including the wild type and the two single-site mutants with higher crystal-packing propensity (A138I and A138Y), yielded three independent crystal forms. Importantly, the condition for one construct did not provide analyzable crystals for the other constructs, indicating that the substitution of a surface residue that increases the crystal-packing propensity successfully produced new crystal-packing interfaces. In addition, because Ala138 is located close to a crystal contact in the wild-type crystal, the introduction of larger amino acids at this position may interfere with the wild-type crystal contact, thereby facilitating crystallization in other forms. This may suggest another applicability of our strategy to change the existing crystal-packing mode. The ASA values buried by crystal packing were similar in all three crystal forms (Supplementary Table S6). About 2000 Å^2^ of buried ASA might be necessary to produce analyzable crystals in the *Ph*BCCP system. In terms of buried ASA, the residue at position 138 in the mutant crystals contributes more to crystal packing when compared with that in the wild-type crystal.

Based on the results of our statistical analysis and of our crystallization experiments, we can discuss issues related to the SER method. Firstly, the replacement of a residue with a higher SCE such as lysine with a residue with an equally high SCE such as arginine or glutamine sometimes has a positive effect on crystal contact formation. Secondly, it is not fully understood whether the effectiveness of a mutation depends on the secondary structure of the target residues. We consider that these issues are correlated. This is illustrated by the example reported by Honjo *et al.* (2008 ▸). Using an experiment in which a surface-exposed glutamate was systematically mutated to alanine, valine, leucine, serine or threonine, they demonstrated that the effects of serine or threonine on crystallization improvement are superior to those of alanine or leucine. They mutated Glu81 located at the terminus of the 3_10_-helix of human acidic fibroblast growth factor. The results showed that the crystal-packing propensities of serine and threonine on a helix are higher than those on sheets and coils, which also means that serine and threonine on a helix tend to be involved in crystal contacts, although they argued that the hydrogen-bond formation induced by the side chain of a serine or threonine residue mediated crystal contact formation. This interesting example is well explained by our crystal-packing propensity of amino acids. In addition, using malate synthase G from *Escherichia coli*, Anstrom and coworkers showed that glutamine is more effective for improvement of crystallization than alanine as a residue to replace lysine. Our results show that the crystal-packing propensity of glutamine is higher than that of alanine for three structure classes, although they concluded that the effect of the mutation of residues on crystal contact formation did not vary depending on secondary structure (Anstrom *et al.*, 2005 ▸). Therefore, we assume that our approach, which considers the crystal-packing propensity of amino acids depending on secondary-structure classes, is expected to enhance surface-engineering approaches. Our results also show that the factors controlling crystal contact formation include not only SCE but also other factor(s), which might include SCE in some sense. According to the multiple linear regression analysis used to identify factors underlying crystal contact formation propensity, the necessary elements controlling crystal contact formation were identified as the hydrophobicity and the side-chain size of residues, rather than the SCE alone. Indeed, the correlation of these factors with the SCE is higher than that of other factors (see Supporting Information). The results of subsequent multiple linear regression analyses clarified a difference in the propensity of residues depending on their secondary structure. This fact indicates that we can suggest different mutation guides that are optimal for residues according to the secondary structure in which the target residue is present.

In our approach, as well as in the SER strategy, the improvement of protein crystallizability by mutation has a common prerequisite that the introduced mutation does not perturb the parent protein structure. The SER strategy may concentrate on coil regions to avoid such perturbations. However, in this work on the *Ph*BCCP system the mutation introduced did not affect the overall structure (Fig. 4 ▸), indicating that a β-sheet can accommodate a successful mutation if it is carefully selected so as to avoid intramolecular steric hindrance. Therefore, the approach presented here involving the substitution of surface residues to increase the crystal-packing propensity is regarded as particularly useful for improving the quality of crystals of known structures, although its applicability should be evaluated using many other proteins with poor crystallizability in the future. Currently, we are developing an effective experimental method to find appropriate surface lysine residues to be substituted (manuscript in preparation), which further enhances the applicability of our approach, including to proteins of unknown structure. In this study, we found an improvement of the crystallizability of *Ph*BCCP that was proportional to the crystal-packing propensity of each amino acid; it produced many non-analyzable crystals (see §[Sec sec2.6]2.6 for definitions) that should be improved by other methods such as crystal contact engineering (Mizutani *et al.*, 2008 ▸) to make them analyzable. From another point of view, the number of analyzable proteins may increase by combining our approach and recent advanced technologies such as X-ray free-electron lasers (FELs), with which nanometre-size crystals can be analyzed (Barends *et al.*, 2014 ▸). This combination of approaches may expand the versatility of the X-ray diffraction method to promote the advancement of protein science.

## Supplementary Material

PDB reference: *Ph*BCCPΔN79, wild type, 5gu8

PDB reference: A138I mutant, 5gu9

PDB reference: A138Y mutant, 5gua

Supporting Information.. DOI: 10.1107/S2059798317010932/wa5114sup1.pdf

## Figures and Tables

**Table 1:** Crystallization screening of *Ph*BCCPΔN79 The number of crystallization conditions used was 528 in total. Microscopic images of crystallization drops with unique conditions at six weeks after the start of the experiment were classified into four categories as described in §[Sec sec2.6]2.6 and counted. Columns are sorted in descending order of packing propensity (sheet) for residue 138 as shown in the last row.

	A138I	A138Y	A138R	A138Q	A138V	Wild type	A138K
Clear drop	379	448	480	449	456	454	472
Amorphous precipitate	4	1	9	20	17	20	30
Non-analyzable crystal	128	73	37	57	53	52	25
Analyzable crystal	17	6	2	2	2	2	1
Packing propensity (sheet)	1.2232	1.1413	1.1171	1.0064	1.0056	0.8875	0.7829

**Table 2:** Data-collection and refinement statistics for the *Ph*BCCPΔN79 crystals Values in parentheses are for the outermost shell.

Crystal	Wild type	A138I	A138Y
Data collection			
Space group	*P*2_1_2_1_2_1_	*C*222_1_	*P*3_1_21
Unit-cell parameters (Å)	*a* = 26.86, *b* = 39.95, *c* = 59.64	*a* = 41.47, *b* = 77.44, *c* = 39.27	*a* = *b* = 39.06, *c* = 76.64
Resolution range (Å)	33.2–1.8 (1.83–1.80)	38.7–1.9 (1.93–1.90)	33.8–1.5 (1.53–1.50)
Completeness (%)	100.0 (100.0)	98.8 (99.1)	99.4 (98.2)
〈*I*/σ(*I*)〉	30.5 (11.4)	47.7 (24.3)	41.2 (16.0)
*R*_merge_ † (%)	6.4 (22.9)	5.9 (9.6)	4.4 (10.2)
Refinement			
Resolution range (Å)	33.2–1.8 (2.27–1.80)	36.6–1.9 (2.39–1.90)	33.8–1.5 (1.65–1.50)
*R*_cryst_/*R*_free_ ‡ (%)	16.0 (15.4)/19.7 (19.5)	16.0 (15.7)/20.6 (25.2)	18.5 (19.6)/20.4 (21.7)
R.m.s.d., bond lengths (Å)	0.009	0.006	0.010
R.m.s.d., bond angles (°)	1.23	1.13	1.28

† *R*_merge_ = 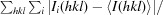 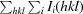, where *I_i_*(*hkl*) is the *i*th observation of reflection *hkl* and 〈*I*(*hkl*)〉 is the weighted average intensity for all *i* observations of reflection *hkl*.

‡ *R*_cryst_ = 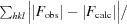 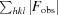, where |*F*_obs_| and |*F*_calc_| are the observed and calculated structure-factor amplitudes, respectively. *R*_free_ was calculated with 5% of the reflections, which were chosen at random and omitted from refinement.

**Table 3:** Crystal-packing interactions in *Ph*BCCPΔN79 crystals Interactions are classified into four types and counted: nonpolar interactions with a distance not greater than 4.0 Å, hydrogen bonds with a distance not greater than 3.4 Å (angle considered), indirect polar interactions mediated by a water molecule or a metal ion, with distances not greater than 3.4 Å (angle considered), and electrostatic interactions with a distance not greater than 4.0 Å. Interatomic distances were calculated using the program *ACT* from the *CCP*4 suite. Values in parentheses denote the number of interactions divided by the total buried ASA on crystal packing given in Supplementary Table S6.

Crystal	Nonpolar interaction	Hydrogen bond	Indirect polar interaction	Electrostatic interaction attractive/repulsive
Wild type	222 (0.108)	18 (0.009)	35 (0.017)	4/0
A138I	232 (0.099)	16 (0.007)	27 (0.012)	14/5
A138Y	275 (0.135)	13 (0.006)	16 (0.008)	4/15
