# Supplementary material for: Designing better diffracting crystals of biotin carboxyl carrier protein from Pyrococcus horikoshii by a mutation based on the crystal-packing propensity of amino acids
Source: Acta Crystallogr D Struct Biol. 2017 Aug 15;73(Pt 9):757–66. doi: 10.1107/S2059798317010932 (PMC5586248; doi:10.1107/S2059798317010932)
Supplement: Supplementary file 1 [file d-73-00757-sup1.pdf]

STRUCTURAL
BIOLOGY

Volume 73 (2017)

Supporting information for article:

Designing better diffracting crystals of biotin carboxyl carrier protein from *Pyrococcus horikoshii* by a mutation based on crystal-packing propensity of amino acids

Kazunori D. Yamada, Naoki Kunishima, Yoshinori Matsuura, Koshiro Nakai, Hisashi Naitow, Yoshinori Fukasawa and Kentaro Tomii

Supporting information for “Designing better diffracting crystals of biotin carboxyl carrier protein from *Pyrococcus horikoshii* by a mutation based on crystal-packing propensity of amino acids”

Kazunori D. Yamada[a, b, 1], Naoki Kunishima[c, 1, 2], Yoshinori Matsuura[c], Koshiro Nakai[c], Hisashi Naitow[c], Yoshinori Fukasawa[a], and Kentaro Tomii[a, d, 2]

[a]Artificial Intelligence Research Center, National Institute of Advanced Industrial Science and Technology (AIST), 2-4-7 Aomi, Koto-ku, Tokyo 135-0064 Japan, [b]Graduate School of Information Sciences (GSIS), Tohoku University, 6-3-09 Aramaki-Aza-Aoba, Aoba-ku, Sendai, Miyagi 980-8579 Japan, [c]RIKEN SPring-8 Center, 1-1-1 Kouto, Sayo-cho, Sayo-gun, Hyogo 679-5148 Japan, [d]Biotechnology Research Institute for Drug Discovery, National Institute of Advanced Industrial Science and Technology (AIST), 2-4-7 Aomi, Koto-ku, Tokyo 135-0064 Japan

[1]Authors contributed equally to this work
[2]Corresponding authors: Naoki Kunishima (kunisima@spring8.or.jp) and Kentaro Tomii (k-tomii@aist.go.jp)

Contents

S1. Crystal-Packing Propensity of Amino Acids
S2. Factors Influencing Crystal-Packing Propensity
S3. Analysis of Accessible Surface Area of Crystal Structures
References
Figure Legends
Tables

S1 Crystal-Packing Propensity of Amino Acids

We calculated the crystal-packing propensity L_i for 20 amino acids (i = 1, 2, ..., 20) from the crystal structure dataset, which was newly compiled from previous reports as described in **Methods** of the main text. The crystal-packing propensity represents the likelihood of involvement of an amino acid of interest in crystal contacts. Figure S3 presents the crystal-packing propensity for each amino acid irrespective of secondary structural classes. By comparing the propensities of each residue, results showed that the rarest residue in the crystal contact interface was lysine. Lysine tends to avoid involvement in crystal contact. However, alanine, the most commonly used residue as a replacement residue of mutation in the SER method, did not have such high propensity: the crystal-packing propensity of alanine was about 0.964.

To confirm our findings of crystal-packing propensities, especially in the secondary structure dependency, we also calculated the crystal-packing propensity using subsets selected randomly from the crystal structure dataset. In this trial, the crystal-packing propensities for 20 amino acids depending on three secondary structural classes and on entire classes were calculated 200 times on every 500 randomly selected entries. Figure S1 depicts boxplots of calculated values of crystal-packing propensities for 20 amino acids depending respectively on three secondary structural classes. We performed Kruskal–Wallis one-way analysis of variance, which revealed that significant differences among bootstrap averages of propensity of each three secondary structure for all amino acids respectively (p<0.01 for all cases). General trends and mean values are consistent with the results presented in Fig. 1B of the main text. In most cases, we found significant differences of crystal-packing propensity of an amino acid depending on secondary structural classes. We infer that these results support our argument in the main text.

S2 Factors Influencing Crystal-Packing Propensity

To identify factors affecting the crystal contact formation, we conducted a multiple linear regression analysis by stepwise forward selection method with the crystal-packing propensity as the explained variable and 10 independent indices, which represent physicochemical properties of amino acids, as candidates for explanatory variables. We used values introduced by Kidera et al. (1) that represent 10 mutually independent physicochemical properties derived from the set of amino acid indices used at that time by application of factor analysis. The set of values defines the important properties of 20 amino acids. The properties include 10 properties such as helix/bend preference, side-chain size, extended structure preference, hydrophobicity, double-bend preference, partial specific volume, flat extended preference, occurrence in alpha region, pK-C, and surrounding hydrophobicity. We conducted multiple linear regression analysis against the crystal-packing propensity for each secondary structure class to pursue a more concrete source of the differences described above. In advance of the regression analysis, the crystal-packing propensity was standardized using the following equation:

$$SL_i = \frac{L_i - \mu}{\sigma}.$$

In that equation, μ and σ respectively represent the mean and sample standard deviation of the crystal-packing propensity. The set of values for the 10 properties had already been standardized similarly. In addition, the values for side-chain entropy (SCE) (2) were standardized in the same manner when we used them for multilinear regression analysis.

Results show that the dominant properties of statistical significance against the crystal-packing propensity on the helix and coil region were hydrophobicity and the side-chain size of residues, which were identical to the case of the propensity on all regions (Table S4). The most dominant properties against the crystal-packing propensity on the sheet region included extension of the structure preference in addition to the two factors. The extended structure preference of isoleucine is quite high, perhaps reflecting the higher value of isoleucine in the crystal-packing propensity. Furthermore, although we conducted the same analyses with the 10 properties and SCE as candidates for explanatory variables, the result did not change (SCE was not sufficient to explain the crystal contact formation likelihood). Therefore, the result suggests that the dominant properties for the crystal-packing propensity are hydrophobicity and the side-chain size of residues, rather than SCE. Nevertheless, we do not completely reject the effect of SCE on crystallization improvement. Rather, although these two factors differed from SCE, probably the two factors partially included the property of SCE because, as shown in Table S5, the correlation coefficient of hydrophobicity and side-chain size with SCE were the first and second highest values among the 10 factors, where the correlation coefficient of hydrophobicity and side-chain size with SCE were, respectively, 0.599 and 0.577. From the collected results of this study, we inferred that the most dominant factors controlling crystallization are hydrophobicity and side-chain size of residues, which might latently include the property of SCE.

S3 Analysis of Accessible Surface Area of Crystal Structures

Crystal packing of the wild type and the two mutants (A138I, and A138Y) was investigated further through analysis of the accessible surface area (ASA). In all three crystal forms, the crystal packing buries the ASA of protein molecule in the asymmetric unit to a similar degree by about 2000 $Å^2$ sharing 47–52% of total ASA (Table S6). As expected, the contribution of the 138th side-chain to the buried ASA was substantially greater in mutants: 3.8% in A138I and 4.9% in A138Y, but only 0.7% in the wild type. A surface representation of the crystal packing interface involving the 138th residue depicts an extensive contribution of the 138th side chain to the crystal-packing interaction (Fig. S2). It is noteworthy that the order of the buried ASA does not correspond to that of the crystal quality in terms of the ability to diffract X-rays.

References

1. Kidera A, Konishi Y, Oka M, Ooi T, Scheraga H (1985) Statistical analysis of the physical properties of the 20 naturally occurring amino acids. *Journal of Protein Chemistry* 4(1):23-55.
2. Doig AJ, Sternberg MJ (1995) Side-chain conformational entropy in protein folding. *Protein Sci* 4(11):2247-2251.

Figure S1

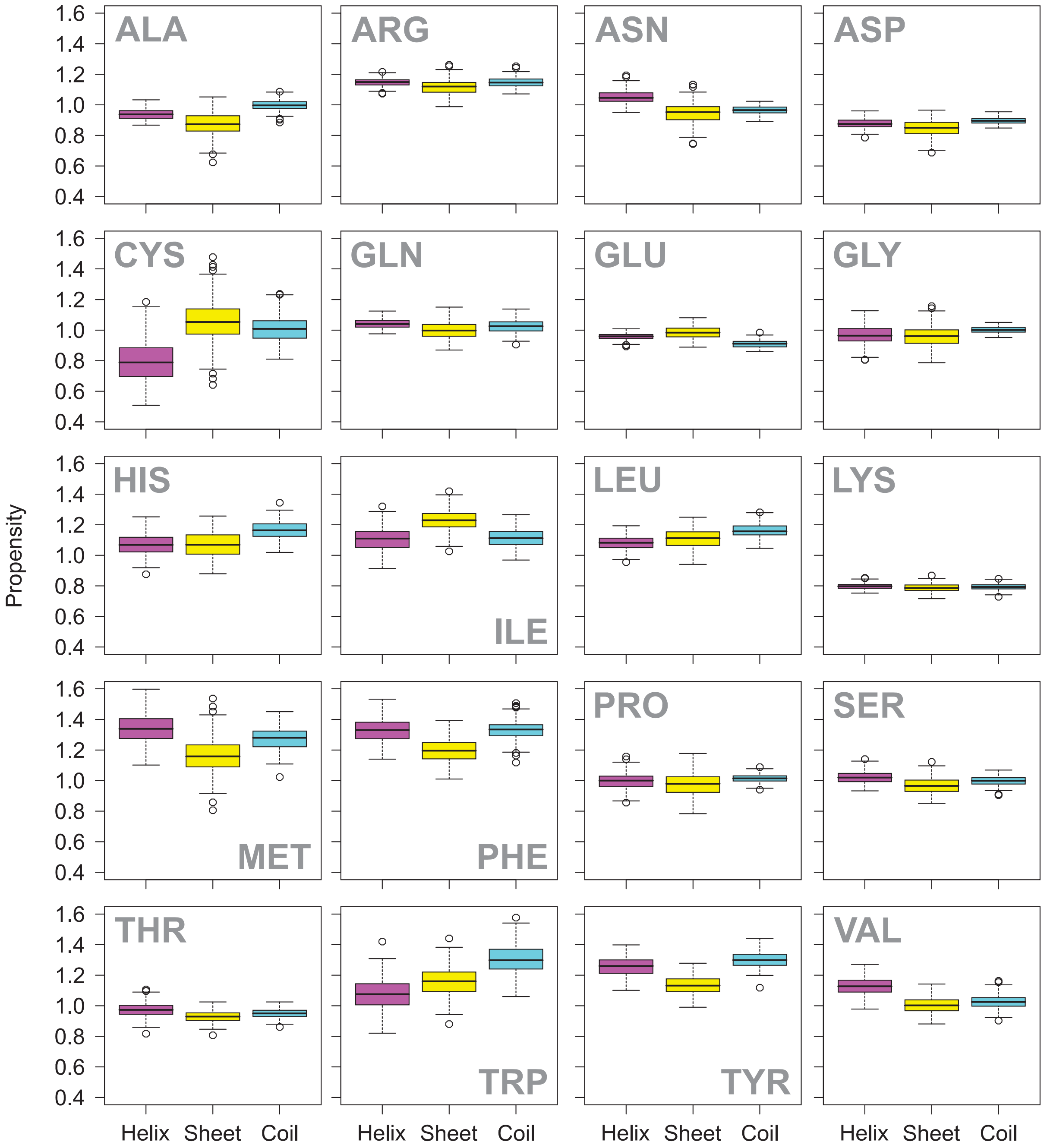

Crystal-packing propensity for each amino acid: Crystal-packing propensities for 20 amino acids depending on three secondary structural classes were calculated 200 times for every 500 randomly selected entries. Boxplots show calculated values of crystal-packing propensities for 20 amino acids. Boxplots show calculated values of crystal-packing propensities for 20 amino acids. The box indicates the range from the lower quartile to the upper quartile. The solid line shows the median. The lower and upper whiskers indicate 1.5 times the interquartile range from the top or bottom of each box, respectively. Data points depicted by circles are outliers.

Figure S2

(*a*)

(*b*)

(*c*)

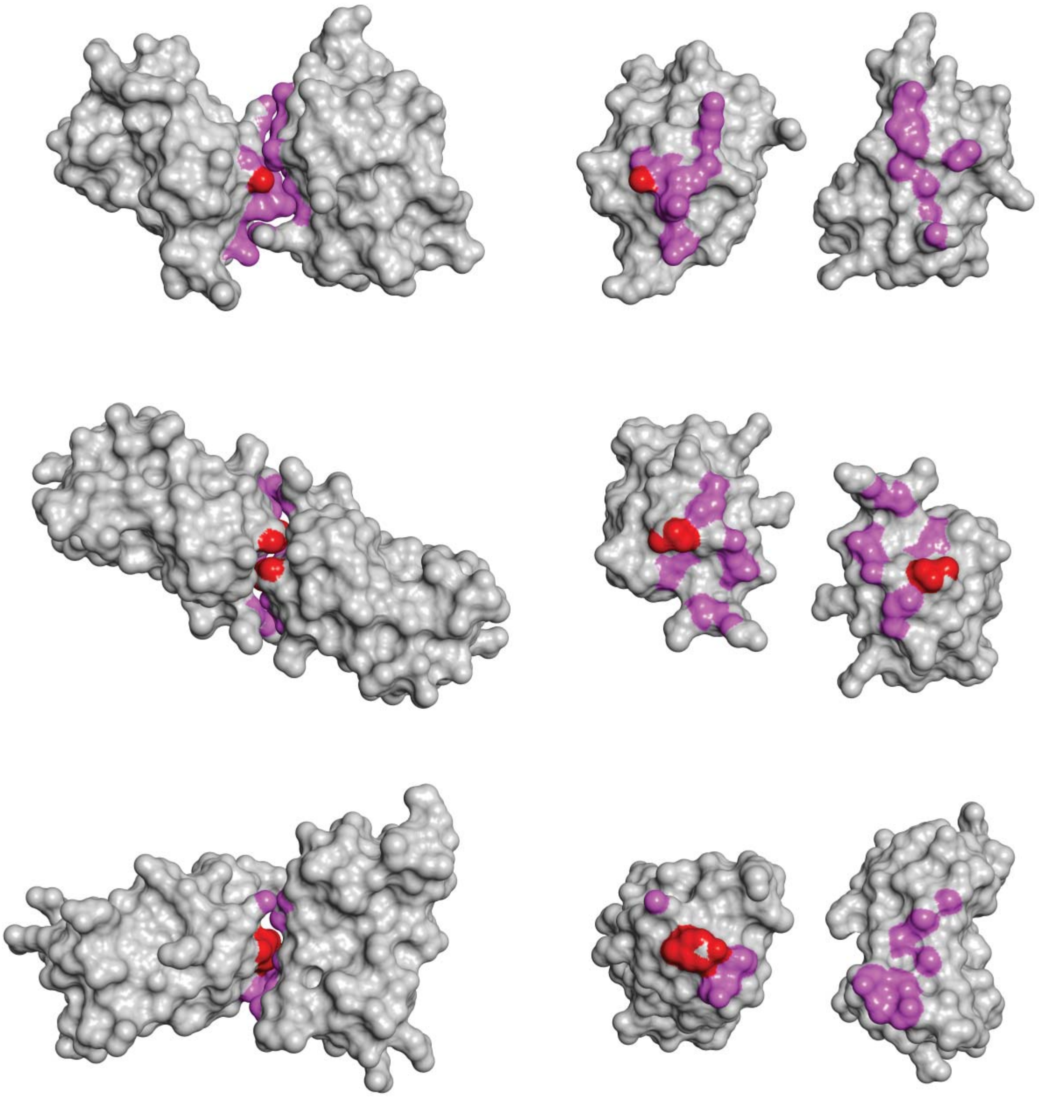

Open-book view of crystal packing interface of *Ph*BCCPΔN79 crystals: wild type (*a*), A138I mutant (*b*), A138Y mutant (*c*). A pair of monomers involving the 138th residue of asymmetric unit is selected. Molecular surfaces for the molecules are calculated with the probe radius of 1.4 Å and displayed as a crystallographic dimer at the left side and its open-book monomers at the right side. Atoms with intermolecular distances not greater than 4.0 Å are colored: side chain atoms of 138th residue in red and others in pink. This figure was prepared with Discovery Studio (Accelrys Inc.).

Figure S3

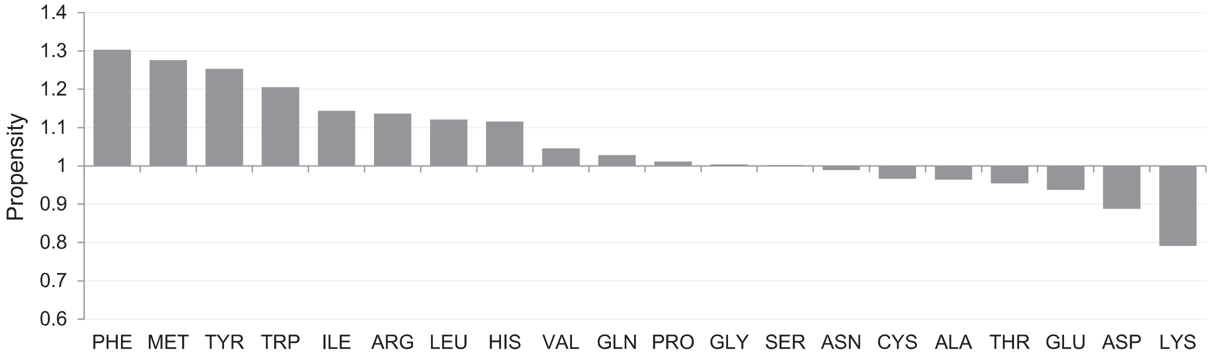

Crystal-packing propensity for each amino acid: Crystal-packing propensities for 20 amino acids for entire classes are shown.

Figure S4

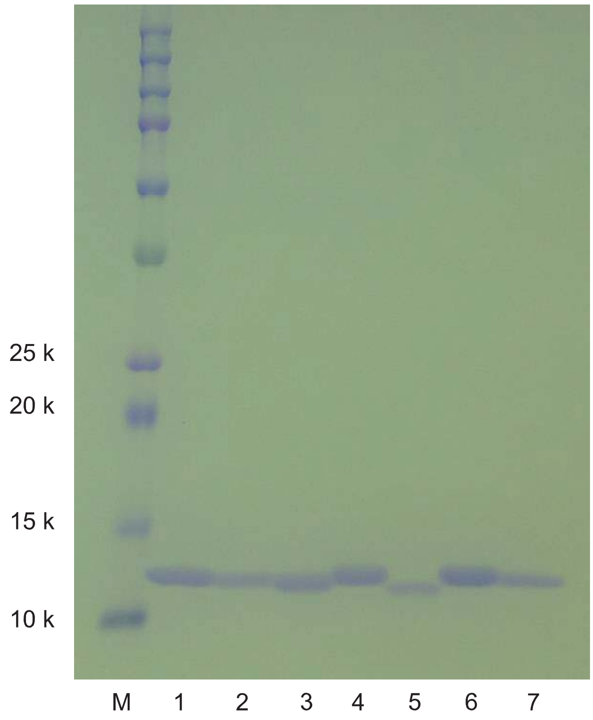

SDS-PAGE of purified *Ph*BCCPΔN79 samples. Proteins were denatured in SDS-PAGE sample buffer, separated on a 12.5% polyacrylamide gel, and stained with Coomassie Brilliant Blue. The quantity of sample applied was 0.6–0.7 μg per lane. Contents: lane M, molecular weight markers with labeling; lane 1, wild type; lane 2, A138I mutant; lane 3, A138K mutant; lane 4, A138Q mutant; lane 5, A138R mutant; lane 6, A138V mutant; lane 7, A138Y mutant.

Table S1. Fisher's exact probability test on crystallization results

P = 0.0286	Rate of amorphous precipitate		Total
	< 2.5%	≥ 2.5%	
Packing propensity (sheet) > 1.04	3	0	3
Packing propensity (sheet) ≤ 1.04	0	4	4
Total	3	4	7

This contingency table was produced based on the corresponding plot in Fig. 2*a*.

Table S2. Details of data collection and refinement

Crystal	Wild type	A138I	A138Y
Data collection			
Space group	$P2_12_12_1$	$C222_1$	$P3_121$
Unit-cell parameters (Å)	*a*=26.86, *b*=39.95, *c*=59.64	*a*=41.47, *b*=77.44, *c*=39.27	*a*=*b*=39.06, *c*=76.64
No. of chains in asymmetric unit	1	1	1
Matthews coefficient	2.08	2.04	2.17
Resolution range (Å)	33.2−1.8 (1.83−1.80)	38.7−1.9 (1.93−1.90)	33.8−1.5 (1.53−1.50)
No. of unique reflections	6329 (288)	5180 (222)	11 290 (613)
Redundancy	6.9 (6.7)	5.8 (4.4)	9.9 (6.1)
Completeness (%)	100.0 (100.0)	98.8 (99.1)	99.4 (98.2)
$\langle I/\sigma(I)\rangle$	30.5 (11.4)	47.7 (24.3)	41.2 (16.0)
[a] R_{merge} (%)	6.4 (22.9)	5.9 (9.6)	4.4 (10.2)
Wilson *B* value (Å^2)	11.3	13.5	10.4
Refinement			
Resolution range (Å)	33.2−1.8 (2.27−1.80)	36.6−1.9 (2.39−1.90)	33.8−1.5 (1.65−1.50)
No. of reflections	6329 (3080)	5178 (2547)	11 290 (2761)
[b] R_{cryst} / R_{free} (%)	16.0 (15.4) / 19.7 (19.5)	16.0 (15.7) / 20.6 (25.2)	18.5 (19.6) / 20.4 (21.7)
[c] Estimated coordinate error (Å)	0.14	0.16	0.10
All model $\langle B\rangle$ (Å^2) / no. of atoms	16.9 / 626	19.6 / 684	17.8 / 657
Non-solvent $\langle B\rangle$ (Å^2) / no. of atoms	15.1 / 540	17.5 / 567	15.9 / 552
Solvent $\langle B\rangle$ (Å^2) / no. of atoms	27.6 / 86	30.1 / 117	27.4 / 105
Rmsd bond lengths (Å)	0.009	0.006	0.010
Rmsd bond angles (º)	1.23	1.13	1.28
Ramachandran plot (%)			
Most favored	94.7	91.2	93.0
Additional allowed	5.3	8.8	7.0
Generously allowed	0.0	0.0	0.0
Disallowed	0.0	0.0	0.0
PDB code	5GU8	5GU9	5GUA

[a] $R_{merge} = \sum_{hkl} \sum_i |I_i(hkl) - \langle I(hkl)\rangle| / \sum_{hkl} \sum_i I_i(hkl)$, where $I_i(hkl)$ is the *i*th observation of reflection *hkl* and $\langle I(hkl)\rangle$ is the weighted average intensity for all observations *i* of reflection *hkl*. [b] $R_{cryst} = \sum_{hkl} ||F_{obs}| - |F_{calc}|| / \sum_{hkl} |F_{obs}|$, where $|F_{obs}|$ and $|F_{calc}|$ respectively denote the observed and calculated structure–factor amplitudes. R_{free} was calculated with 5% of the reflections chosen at random and omitted from refinement. [c] Maximum-likelihood based value from the program PHENIX (Adams et al., 2010). Values in parentheses are for the outermost shell.

Table S3. Superposition of coordinates between *Ph*BCCPΔN79 crystals

C^{α} 79–149: 71 atoms	Wild type	A138I	A138Y
Wild type	-	1.43 Å	1.40 Å
A138I	-	-	1.21 Å
A138Y	-	-	-

C^{α} 81–149: 69 atoms	Wild type	A138I	A138Y
Wild type	-	0.57 Å	0.35 Å
A138I	-	-	0.49 Å
A138Y	-	-	-

Main chain 81–149: 276 atoms	Wild type	A138I	A138Y
Wild type	-	0.53 Å	0.37 Å
A138I	-	-	0.49 Å
A138Y	-	-	-

Side chain 81–149: 243 atoms	Wild type	A138I	A138Y
Wild type	-	1.65 Å	1.63 Å
A138I	-	-	1.52 Å
A138Y	-	-	-

All 81–149: 519 atoms	Wild type	A138I	A138Y
Wild type	-	1.21 Å	1.16 Å
A138I	-	-	1.11 Å
A138Y	-	-	-

Superposition of corresponding atoms was performed on all pairs of the *Ph*BCCPΔN79 crystals using the program LSQKAB of the CCP4 suite. The r.m.s.d. values of the interatomic distances between corresponding atoms after the superposition are shown. Selected atoms used for the superposition are presented at the top left corner in each table.

Table S4. Results of multiple linear regression analysis

	β	*p*-value
All		
Hydrophobicity	-0.594	0.00104**
Side-chain size	0.487	0.00447**
Extended structure preference	0.223	0.149
Occurrence in alpha region	0.188	0.217
Helix		
Side-chain size	0.438	0.0378*
Hydrophobicity	-0.370	0.0736
Extended structure preference	0.266	0.188
Sheet		
Hydrophobicity	-0.590	0.000508**
Extended structure preference	0.343	0.0218*
Side-chain size	0.460	0.00368**
Partial specific volume	0.227	0.110
Coil		
Hydrophobicity	-0.637	0.000182**
Side-chain size	0.535	0.000944**

β indicates the slope for each variable and *p*-values for the results of t-test, where the null hypothesis is that value of the slope will be zero, are shown ($^{*}p<0.05$ and $^{**}p<0.01$).

Table S5. Correlation coefficients between physicochemical properties and SCE

Kidera property factor	Correlation to SCE
Helix/bend preference	-0.220
Side-chain size	0.577
Extended structure preference	0.139
Hydrophobicity	0.599
Double-bend preference	0.103
Partial specific volume	0.217
Flat extended preference	0.130
Occurrence in alpha region	-0.113
pK-C	-0.128
Surrounding hydrophobicity	-0.0390

The Pearson correlation coefficient between SCE and each variable was calculated.

Table S6. Accessible surface area analysis of *Ph*BCCPΔN79 crystals

Crystal	ASA (Å^2)	Buried ASA by crystal packing (Å^2)	
		Total	Sidechain 138th
Wild type	4409	2058	14
A138I	4480	2339	90
A138Y	4360	2041	99

Accessible surface area (ASA) was calculated using the program SURFACE in the CCP4 suite.
